# GDNF neurotrophic factor signalling determines the fate of dermal fibroblasts in wound‐induced hair neogenesis and skin regeneration

**DOI:** 10.1111/exd.14526

**Published:** 2022-01-22

**Authors:** Neda Vishlaghi, Sandra Rieger, Vanessa McGaughey, Thomas S. Lisse

**Affiliations:** ^1^ 5452 Biology Department Cox Science Center University of Miami Coral Gables Florida USA; ^2^ 5452 Sylvester Comprehensive Cancer Center Miller School of Medicine University of Miami Miami Florida USA

**Keywords:** fibroblasts, GDNF, GFRA1, hair follicles, regeneration, RET, skin, stem cells, wound healing, wound repair

## Abstract

We propose that GDNF, a glial cell line‐derived neurotrophic factor, can promote hair follicle neogenesis and skin regeneration after wounding by directing the fate of dermal fibroblasts. Our hypothesis is largely based on detailed GDNF and receptor analysis during skin regenerative stages, as well as the induction of GDNF receptors after wounding between the pro‐regenerative spiny mouse (genus *Acomys*) and its less‐regenerative descendant, the house mouse (*Mus musculus*). To characterize the GDNF‐target cells, we will conduct a series of lineage‐tracing experiments in conjunction with single‐cell RNA and assay for transposase‐accessible chromatin sequencing experiments. The heterogenetic dynamics of skin regeneration have yet to be fully defined, and this research will help to advance the fields of regenerative medicine and biology. Finally, we believe that stimulating the GDNF signalling pathway in fibroblasts from less‐regenerative animals, such as humans, will promote skin regeneration, morphogenesis and scarless wound healing.

## 1 BACKGROUND

Because the nervous system and the skin epidermis share an ectodermal origin, neurotrophic factors may play critical roles in controlling skin appendage formation and homeostasis.^1^, ^2^, ^3^, ^4^ Glial cell line‐derived neurotrophic factor (GDNF), a well‐studied neuroprotective factor,^5^, ^6^ has recently been identified as a neurotrophic factor that promotes the formation of hair follicles in mice.^7^ The GDNF family of ligands (which includes neurturin [NRTN], artemin [ARTN] and persephin [PSPN]) mediate RET tyrosine kinase activation via a ligand‐binding receptor subunit called GDNF factor receptor alpha (GFRA).^8^ There are four GFRA family members, with GDNF preferentially binding to GFRA1, NRTN, ARTN and PSPN binding to GFRA2, GFRA3 and GFRA4 respectively.^9^ Furthermore, in cells lacking RET, neural adhesion molecule (NCAM) can directly interact with GFRA1‐GDNF to regulate cell–cell communication,^10^, ^11^, ^12^ which has yet to be investigated in skin cells.

**FIGURE 1:**
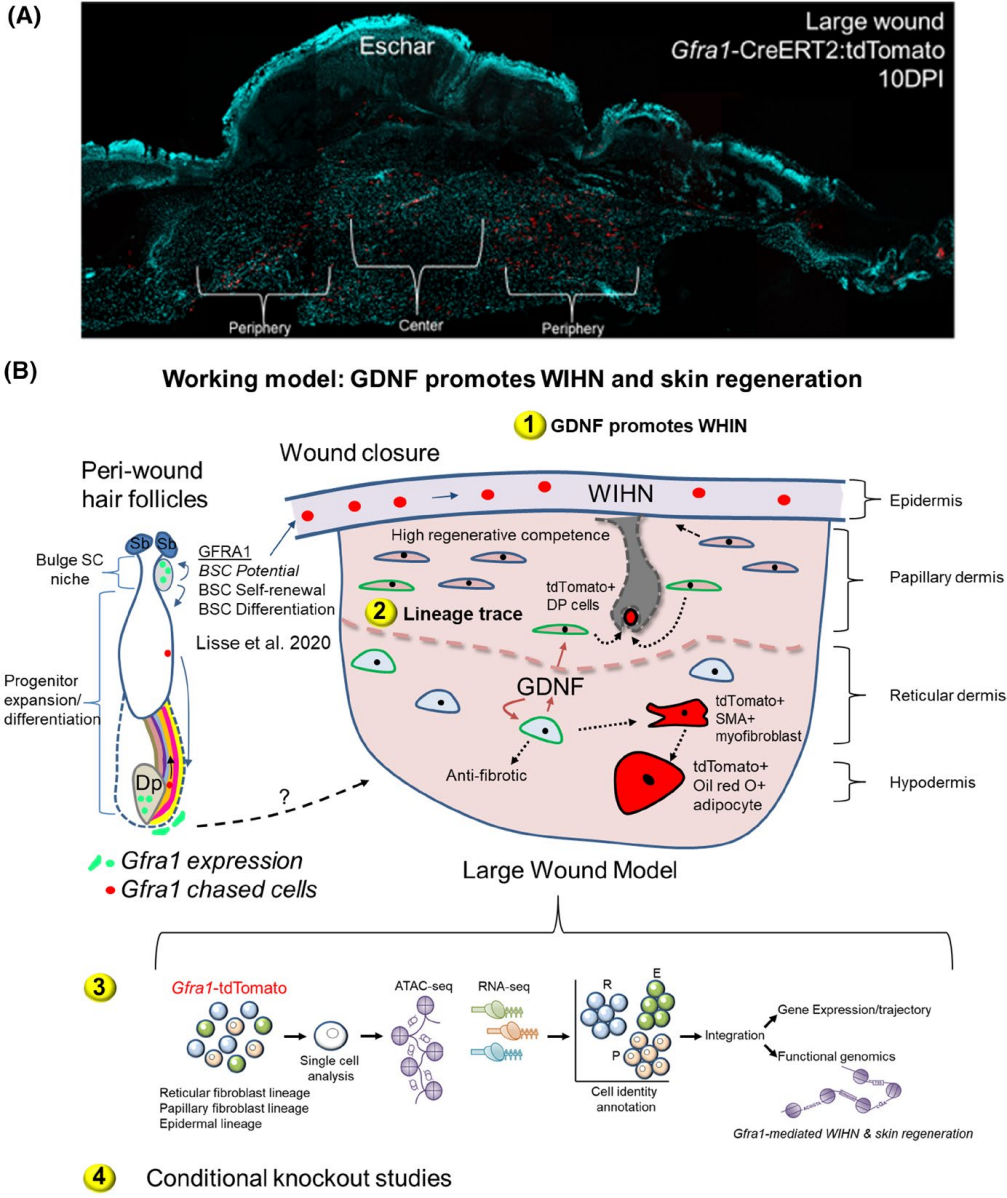
GDNF‐GFRA1 signalling promotes hair and skin regeneration. (A) *Gfra1*‐positive cells contribute to large wounds. Short‐term lineage tracing was performed using *Gfra1*‐CreERT2:tdTomato reporter mice subjected to large wounds. Wound sections represent wound healing at 10 DPI using tiled confocal images (Zeiss Airyscan). Representative wound section shown (*n* = 3 experiments performed). DAPI (nuclei). (B) Proposed working model of how GDNF can promote WIHN and skin regeneration in mice. The numbers correspond to hypothesis testing (Section 4). *Left*. GFRA1 is expressed by BSCs and dermal papillary (Dp) and sheath cells (marked Green). Previous lineage‐tracing studies showed that GDNF signalling can specify BSCs to the epidermal and hair follicle lineages depending on tissue environment conditions (marked in Red). Sebaceous glands (Sb). *Right*. *Gfra1* is expressed in distinct populations of dermal fibroblasts during wound healing of large wounds (marked with Green outline). (1) GDNF‐GFRA1 signalling may regulate wound healing and appendage regeneration by targeting both papillary and reticular dermal fibroblasts. (2) This may occur at the expense of committing regeneration‐competent papillary fibroblasts to the Dp lineage to support the hair neogenesis process. In addition, GDNF may promote the differentiation of GFRA1+ reticular dermal fibroblasts into SMA‐expressing myofibroblasts, and then subsequent reprogramming of myofibroblasts into fat cells to support the wound environment. (3) Schematic overview of the sequencing workflow. Combined scRNAseq and scATACseq will be applied to identify and define the cell transition states and the drivers of such trajectories in the large‐wound model. (4) Conditional *Gfra1* knockout strategies will be applied to determine the functional requirements of *Gfra1* signalling within distinct subsets of dermal fibroblasts and *Gfra1*‐expressing cells

Previous research has found that *Gdnf* expression correlates with different stages of the natural hair cycle, and that both GDNF and NRTN can control the murine hair cycle, as loss of function of both *Gfra1* and *Gfra2* results in increased regression of hair follicles.^4^ Our group demonstrated that *Gfra1* is specifically expressed by dermal papillary (DP) cells and bulge stem cells (BSC) of hair follicles using a *Gfra1* gene reporter mouse line.^7^ (Figure 1). DP cells are the mesenchymal component of hair follicles that control the activation of adult BSCs at rest and the differentiation of actively proliferating progenitor cells committed to the hair follicle lineage.^13^, ^14^ Furthermore, DP cells can induce epithelia to form hair follicles and may have stem cell‐like properties because they can reconstitute the skin dermis.^14^, ^15^ BSCs are multipotent, and lineage commitment occurs when they are directed to become epidermal, hair follicle or sebaceous gland cells—an important function for correcting any imbalances that may occur during injury and/or disease.^16^ From the standpoint of hair follicle stem cells, it is unknown whether *Gfra1* can specify a lineage. Furthermore, whether *Gfra1* can specify the fate of dermal fibroblasts to reconstitute and repattern damaged dermis is unknown from a wound healing and regenerative standpoint. Answering these questions is a significant step forward in hair biology, as it connects a major neurotrophic factor to both skin homeostasis and regeneration.

Several species, including zebrafish and axolotls, can overcome scarring via epimorphic regeneration, a process similar to embryonic tissue development in which less differentiated blastemal cells emerge and retain positional memory to form new tissues.^17^, ^18^, ^19^ Mammalian species, on the contrary, do not typically regenerate lost/damaged cutaneous tissue; instead, damaged tissues are replaced by a dense, fibrotic scar.^20^, ^21^ Given the presence of conserved genes in species with high‐ and low‐regenerative capacity, it is likely that less‐regenerative organisms have the ability to regenerate tissues and organs if provided with the appropriate lineage‐specific factors and induction of conserved gene regulatory programs.^22^, ^23^ Interestingly, some mammalian species, such as the African/Egyptian spiny mouse (genus *Acomys*),^24^ have retained the ability to regenerate skin appendages without scars after injury, in contrast to the house mouse, a more recent descendant of the Old‐World mouse lineage (*Mus musculus*).^24^, ^25^ Spiny mice are notable for their ability to regenerate skin through wound‐induced hair neogenesis (WIHN). WIHN does occur in the house mouse, but only in severe wounds and to a much lesser extent.^26^, ^27^, ^28^, ^29^, ^30^, ^31^, ^32^, ^33^, ^34^ During WIHN, a progeny of interfollicular, epidermal and dermal cells become ‘embryonic‐like’ to restore early epithelial‐mesenchymal interactions, resulting in the regeneration of hair follicles, fat and arrector pili muscle.^35^ It is worth noting that the roles that neurotrophic factors play during WIHN have yet to be investigated.

## 2 PREMISE

### 2.1 Scarless wound healing and hair follicle regeneration are associated with increased *Gdnf* and *Gfra1* expression in spiny mice

We re‐examined the gene expression data published by the Maden group, which compared the skin injury responses of house and Egyptian spiny mice (*Acomys cahirinus*).^36^ Adult spiny mice exhibit a WNT‐mediated dermal fibroblast response after wounding, according to the original study. However, we discovered a previously unknown statistically significant fivefold increase in *Gdnf* and *Gfra1* mRNA expression at 7 and 14 days post‐injury (DPI) in spiny mice (*n* = 4, adjusted *p* value range *p* ≤ 0.0001–0.001, two‐way ANOVA with Tukey's multiple comparisons test). Because GFRA1 can be released by cells after injury,^37^ a portion of soluble GFRA1 may influence distant cells to modulate regeneration as well. House mice, on the contrary, showed a statistically significant decrease in *Gdnf* mRNA levels by 14 DPI but no change in *Gfra1* levels. Furthermore, both spiny and house mice showed an insignificant and statistically significant decrease in *Nrtn* and *Gfra2* expression after injury.^38^ There were no statistically significant differences among the other members of the GDNF ligand/receptor family. Finally, after wounding, *Ncam1* was significantly induced (5.5‐fold to 3.7‐fold, respectively, adjusted *p* ≤ value 0.0001) in both house and spiny mice,^36^ implying that co‐induction of *Gfra1* and *Ncam1* may be required for the regenerative phenotype in spiny mice. Overall, these findings imply that Gdnf‐Gfra1 signalling may have a conserved functional role during wound healing and WIHN.

### 2.2 *Gdnf* and *Gfra1* mRNA are expressed in distinct dermal fibroblast populations in house mouse regenerating skin

*Gdnf* was found to be expressed primarily by lower‐repair‐competent dermal fibroblasts, implying the formation of a ‘new’ GDNF‐rich substratum to support the wound environment, similar to how it is seen in early organ development.^39^, ^40^ Furthermore, *Gdnf* and *Gfra1* are expressed by neuronal‐like cells and dermal fibroblasts in neonatal regeneration‐competent skin wounds.^41^ Although older 21‐day‐old animals had *Gfra1* expression in neuronal‐like cells, there was less transcript in dermal fibroblasts than in neonatal wounds.^41^ This suggests that as mice age, the GDNF dermal response decreases, as does the skin's overall regenerative capacity.^42^ Furthermore, a recent transcriptional analysis of developing skin revealed that *Gdnf* and *Gfra1* are expressed by the dermal condensate and sheath cell populations.^43^, ^44^ Global *Gfra1* ablation in neonatal mice resulted in DP cell atrophy and decreased hair follicle survival, according to unpublished research from our laboratory. Thus, adult animal skin appendage regeneration and wound healing may rely on an early fibroblastic reprogramming strategy mediated by GDNF‐GFRA1.

### 2.3 *Gfra1*‐positive cells are found in the dermis of house mice after large skin wounds

Previous research in house mice demonstrated that GDNF improves the outcomes of small non‐regenerative skin wounding, which involves the re‐emergence of embryonic structural factors in the reticular dermis (Lisse et al., 2020). We show the trajectories of *Gfra1*‐positive cells by short‐term lineage‐tracing experiments using the *Gfra1*‐CreERT2:tdTomato mouse model in unpublished studies using the large regenerative wound model in house mice (Figure 1A). Lineage tracing after 10 DPI in these studies revealed the contribution of tdTomato‐positive cells to both the large‐wound centre and the periphery, where the former represents the pool of regeneration‐competent papillary fibroblasts that give rise to the DP cells of neogenic hair follicles (Abbasi et al., 2020; Phan, Sinha, et al., 2020).

## 3 HYPOTHESIS

By directing the fate of dermal fibroblasts, GDNF‐GFRA1 signalling promotes wound‐induced hair neogenesis and skin regeneration.

## 4 HOW TO TEST THE HYPOTHESIS

- At 22 days of age, large‐wound assays (ie ≥1 cm × 1 cm) will be performed in house mice with and without carrier‐free recombinant GDNF, followed by skin regeneration and wound healing assessments for up to 45 days.^28^ Because large wounds have a significant population of *Gfra1*+ dermal fibroblasts at 10 DPI (Figure 1A),^30^ we will inject recombinant GDNF (25 µg/wound; single‐dose) or vehicle into the wound at this timepoint to modulate the underlying dermal fibroblasts. A whole‐mount tissue clearing method will be used to assess qualitative and quantitative analyses of neogenic hair follicles, as well as immunofluorescence and real‐time PCR analyses for markers of early/mature hair follicle development.^45^ Using histological planimetry and polarized light microscopy, we will investigate wound healing and scar integrity.^7^ We will also use immunostaining and Western blotting to examine soluble GDNF gradients, NCAM/RET signalling and post‐translational modifications. Monitoring the subcellular localization of GFRA1 will be used to assess the endocytic receptor‐mediated pathway. Because GFRA1 has the potential to function as a soluble receptor,^37^ we will detect *Gfra1* transcript (RNAscope) and GFRA1 protein simultaneously to identify cells that may function in *trans*.
- Long‐term fate‐mapping studies using the *Gfra1*‐CreERT2:tdTomato reporter mouse model will be used to determine the contribution and identity of GDNF responding cells to WHIN and wound healing. Tamoxifen treatment prior to wounding at 22 days of age will label *Gfra1*‐expressing cells. *Gfra1*‐labelled cells will be studied up to 25–45 DPI to determine their differentiation trajectories. Clonal analysis will be performed between treatment groups,^7^ and the potential for transdifferentiation will be determined using co‐labelling approaches (eg detecting tdTomato‐positive alpha smooth muscle action‐positive myofibroblasts and LipidTOX fat cells).
- We hope to discover novel cellular potential and regulatory networks as part of the GDNF‐GFRA1 signalling pathway by using an integrative scRNAseq and single‐cell assay for transposase‐accessible chromatin sequencing (scATACseq) approach (Figure 1B). To begin, *Gfra1*‐tdTomato‐positive cells will be isolated prior to wounding as well as from dissociated wounds at 10–45 DPI. Purified cells will then be run through the scRNAseq and scATACseq pipelines to obtain sequencing data. Unsupervised bioinformatics analysis will be used to reconstruct *Gfra1*‐mediated trajectories and identify *cis* and *trans*‐regulatory elements as revealed by differential gene expression and chromatin accessibility in individually labelled cells (Heinz et al., 2010).
- Because *Gfra1* global ablation is linked to neonatal lethality,^46^ conditional *Gfra1* deletion studies will be carried out in adult mice to investigate the cellular requirements of GDNF‐GFRA1 signalling during WIHN. To begin, *Gfra1*‐CreERT2:*Gfra1* ^flox/flox^ mice will be studied in large‐wound assays as described in Section 4a to investigate the effects of ablating *Gfra1*‐expressing cells. Finally, conditional ablation of *Gfra1* within *Lrig1*+ cells will be used to assess the functional requirements of *Gfra1* specifically within regeneration‐competent papillary dermal fibroblasts. This will be accomplished by phenotyping *Lrig1*‐CreERT2:*Gfra1* ^flox/flox^ mice in large‐wound assays as described previously.^47^

## 5 RELEVANCE AND PERSPECTIVES

Comparative studies of high‐ and low‐regenerative organisms reveal which pathways to potentially manipulate to promote regeneration. The current regeneration models are insufficient due to a lack of comparative studies. We propose that repurposing the GDNF signalling program found in adult spiny mice is one piece of the regenerative ‘puzzle’ that can be used to build new skin and appendages in less‐regenerative organisms. Testing our evolutionary‐based hypothesis will provide the foundation for future development of GDNF‐based treatment options for impaired wound healing and tissue regeneration caused by diabetes, burns and scarring, which largely reflect functional fibroblasts. Integration of GDNF with biodegradable polymers and extracellular matrix meshes, as well as conjugation with carbon dots, may be used to improve delivery and bioactivity in the wound setting.^48^ Understanding the regulation of GDNF‐based programs and their integration with other specialized programs to collectively enable human regeneration will be the subject of interesting future studies.

## CONFLICT OF INTEREST

The authors have no conflict interests to declare.

## AUTHOR CONTRIBUTIONS

TSL generated the initial ideas, compiled and analysed the data, and wrote the hypothesis letter. NV, SR and VM edited the letter and contributed to intellectual concepts presented in this letter.

## Data Availability

Data available on request from the authors.
